# Frequency of biologic switching and the outcomes of switching in children and young people with juvenile idiopathic arthritis: a national cohort study

**DOI:** 10.1016/S2665-9913(20)30025-4

**Published:** 2020-03-09

**Authors:** Lianne Kearsley-Fleet, Eleanor Heaf, Rebecca Davies, Eileen Baildam, Michael W Beresford, Helen E Foster, Taunton R Southwood, Wendy Thomson, Kimme L Hyrich, BCRD and BSPAR-ETN study groups

**Affiliations:** a Centre for Epidemiology Versus Arthritis, The University of Manchester, Manchester Academic Health Science Centre, Manchester, UK; b Clinical Academic Department of Paediatric Rheumatology, Alder Hey Children's NHS Foundation Trust, Liverpool, UK; c Institute of Translational Medicine (Child Health), University of Liverpool, Liverpool, UK; d Musculoskeletal Research Group, Institute of Cellular Medicine, Newcastle University, Newcastle upon Tyne, UK; e Paediatric Rheumatology, Great North Children's Hospital, Newcastle upon Tyne, UK; f Institute of Child Health, University of Birmingham and Birmingham Children's Hospital, Birmingham, UK; g Centre for Genetics and Genomics Versus Arthritis, Centre for Musculoskeletal Research, Faculty of Biology, Medicine and Health, The University of Manchester, Manchester, UK; h National Institute of Health Research Manchester Biomedical Research Centre, Manchester University NHS Foundation Trust, Manchester Academic Health Science Centre, Manchester, UK

## Abstract

**Background:**

Information is scarce about biological disease-modifying antirheumatic drug (DMARD) switching patterns in children and young people (aged ≤16 years) with juvenile idiopathic arthritis in an era of many biologic therapies. The best choice of biologic to use if the first biological DMARD is not beneficial also remains unclear. We aimed to quantify and characterise biologic switching patterns in children and young people with juvenile idiopathic arthritis, and to compare the effectiveness of using a second tumour necrosis factor inhibitor (TNFi) versus non-TNF is following failure of a first TNFi biologic in routine clinical practice.

**Methods:**

Our study population comprised patients with juvenile idiopathic arthritis who were enrolled in two parallel UK cohort studies (the British Society for Paediatric and Adolescent Rheumatology Etanercept Cohort Study [BSPAR-ETN] and the Biologics for Children with Rheumatic Diseases [BCRD] study) between Jan 1, 2004, and April 11, 2019. Data on disease characteristics and DMARD therapy were collected at the time of initiation of a first biologic, at 6 months, at 1 year, and annually thereafter. Biologic switching patterns were described in all patients who started their first biologic from Jan 1, 2010, onwards. Among patients who started treatment with their first biologic from Jan 1, 2004, onwards, had polyarticular course juvenile idiopathic arthritis (extended oligoarthritis or polyarthritis [positive or negative for rheumatoid factor]), and who had started a second biologic, we assessed changes in outcome variables at 6 months compared with baseline and compared the proportion of patients who achieved an American College of Rheumatology Pediatric (ACR Pedi) 90 response and minimal disease activity at 6 months on the basis of the class of the second biologic (a second TNFi *vs* non-TNFi biologic). Changes in outcome variables at 6 months were compared using linear regression or logistic regression, adjusted for propensity quintiles to account for confounding by indication. We used multiple imputation to account for missing data.

**Findings:**

Between Jan 1, 2004, and April 11, 2019, 2361 patients were enrolled on initiation of biologic therapy. From Jan 1, 2010, onwards, 1152 patients started their first biologic, most of whom started treatment with TNFis (1050 [91%]). The median follow-up was 2·2 years (IQR 1·1–3·8). During this time, 270 (23%) of 1152 patients started a second biologic, 61 (5%) started a third biologic, and 11 (1%) started a fourth biologic. Among 240 patients with polyarticular-course juvenile idiopathic arthritis, 194 (81%) started a second TNFi and 46 (19%) started a non-TNFi after an initial TNFi had failed. Choice of second treatment (second TNFi *vs* non-TNFi biologic) did not affect the proportion of patients who achieved an ACR Pedi 90 response (adjusted odds ratio [OR] 2·5, 95% CI 0·8–7·9; p=0·11) or minimal disease activity (adjusted OR 1·6, 95% CI 0·6–3·8; p=0·33).

**Interpretation:**

For many children and young people with juvenile idiopathic arthritis, treatment with a first or second biologic is not beneficial. We found no evidence that switching to a second non-TNFi biologic was more beneficial than a second TNFi.

**Funding:**

Versus Arthritis and The British Society for Rheumatology.

## Introduction

Biological disease-modifying antirheumatic drugs (DMARDs), or biologics, have become a main treatment option in juvenile idiopathic arthritis, particularly for individuals who do not respond to, or are intolerant of the conventional synthetic DMARDs, such as methotrexate. The introduction of biological DMARDs has improved patient outcomes, and many more children now reach adulthood without substantial joint damage or complications from persistent uveitis compared with the pre-biologic era.^1^, ^2^ Tumour necrosis factor inhibitors (TNFis), such as etanercept and adalimumab, remain the most commonly prescribed biologics for juvenile idiopathic arthritis.^3^ However, several other classes of biological DMARDs are now available, including the T-cell co-stimulatory modulator abatacept, the interleukin (IL)-6 pathway inhibitor tocilizumab, IL-1 inhibitors (including the IL-1 receptor antagonist anakinra and IL-1β inhibitor canakinumab), and the targeted B-cell depleting drug rituximab (not licensed for juvenile idiopathic arthritis). The anti-IL-1 and anti-IL-6 classes of biologics are now considered first-line biologic therapy for children and young people with systemic juvenile idiopathic arthritis.^4^

**Research in context**

**Evidence before this study**

Biological therapies have become a mainstay of treatment for many autoimmune diseases, including juvenile idiopathic arthritis. However, not all children and young people respond to treatment with the first biologic they are prescribed, and the extent to which further exposure to biologics occurs is largely unknown. In 2013, Otten and colleagues described patterns of biologic switching in a Dutch registry of patients with juvenile idiopathic arthritis who started etanercept as their first biologic; however, these patients were recruited before 2010, when few biologic therapies were available. We searched PubMed for studies of biologic therapies in juvenile idiopathic arthritis, published between Jan 1, 2000, and Dec 31, 2019, using the search terms “biologic*”, and “JIA” (or “juvenile” and “arthritis”) and “cohort” or “regist*”. We found no studies assessing the next best choice of biologic if the first biologic (usually a tumour necrosis factor inhibitor [TNFi]) is not beneficial.

**Added value of this study**

This analysis included children and young people with juvenile idiopathic arthritis who were enrolled in one of two UK studies: the Biologics for Children with Rheumatic Diseases study and the British Society for Paediatric and Adolescent Rheumatology Etanercept Cohort Study. Our results showed that biologic switching is not uncommon, with more than one-fifth of patients starting a second biologic and 5% receiving at least three; however, switching often occurred within the same class of biologic rather than between different classes of biologic. The response to a second biologic was similar between patients who switched to a biologic of the same class and those who switched to a biologic of a different class.

**Implications of all the available evidence**

On the basis of these findings, no evidence exists to support or refute the 2015 biologic prescribing guidelines of the National Health Service (NHS) England, which recommend that the majority of patients with juvenile idiopathic arthritis switch to a second TNFi, or the 2019 guidelines of the American College of Rheumatology, which recommend that patients switch to an alternative class of biologic (eg, tocilizumab or abatacept). Repeat analysis with a larger sample size is required to validate these findings. These data will be used to inform practice guidelines, cost-effectiveness, and policy guidelines.

The aim of juvenile idiopathic arthritis treatment is to achieve inactive disease or remission, to enable normal development and growth.^5^ Unfortunately for some individuals, treatment with methotrexate or the first biologic prescribed does not result in disease control or is discontinued due to adverse events. Some children and young people will switch to a second or subsequent biologics until disease control is achieved.^3^ For some individuals, disease control is not achieved despite cycling through multiple biologics. A 2018 publication about adults with rheumatoid arthritis suggested that at least 6% of patients received at least three different classes of biologic and 21% received at least three different biological drugs, often including multiple TNFis.^6^ A Dutch registry of patients with juvenile idiopathic arthritis who started etanercept as a first biologic before 2010 has previously described patterns of biologic switching.^7^ However, these patients were recruited when few biologic therapies were available and thus might now be outdated.

Until 2015, the prescribing of biological DMARDs in the UK was regulated by the National Institute for Health and Care Excellence guidance, which stated that biologics should be reserved for children with juvenile idiopathic arthritis, including systemic juvenile idiopathic arthritis, following previous treatment with methotrexate.^8^, ^9^ In 2015, National Health Service (NHS) England published a new treatment pathway for juvenile idiopathic arthritis, which still recommended treatment with methotrexate before biological therapy, with the exception of individuals with enthesitis-related juvenile idiopathic arthritis, who were recommended to start a TNFi, or individuals with macrophage activation syndrome non-responsive to corticosteroids, who were recommended to initiate treatment with anakinra. After methotrexate, the majority of children with juvenile idiopathic arthritis should start an initial TNFi, except those with systemic juvenile idiopathic arthritis, who are recommended to start tocilizumab. After an initial biologic is ineffective, patients with systemic juvenile idiopathic arthritis can switch to anakinra, whereas all other patients are recommended to start a second TNFi, with the exception of patients who are positive for rheumatoid factor, who are recommended to start rituximab.^5^

With the increasing availability of other biologic classes, the proportion of children and young people with juvenile idiopathic arthritis who are switching between biologics remains unclear. A better understanding of patients with juvenile idiopathic arthritis who switch biologic drugs multiple times is vital to better inform future treatment guidelines and health economic evaluations. Additionally, although it is recognised that children are switching between biologics, no data are available to inform prescribing after patients do not respond to treatment with a first biologic, usually a TNFi. As a result of the paucity of real-world evidence, at present two conflicting recommendations exist for patients when switching to a second biologic due to ineffectiveness of an initial TNFi: 2015 NHS England guidelines^5^ suggest treatment with a second TNFi and 2019 American College of Rheumatology (ACR) guidelines^10^ suggest treatment with a different class of biologic. The use of real-world data to identify the optimum choice of second biologic is methodologically challenging because of the potential of confounding by indication; patients might be prescribed a certain biological therapy due to their characteristics (ie, systemic features, disease severity), and thus comparing the treatments might be confounded by these characteristics. Therefore, careful statistical approaches must be considered.

In this study, we aimed to quantify the proportion of children and young people with juvenile idiopathic arthritis starting a first biologic who subsequently switch biologic therapy, the extent of multiple switching, and with what pattern switching occurs. We also aimed to compare the effectiveness of different classes of biologics after switching from a first TNFi in routine clinical practice.

## Methods

### Study design and participants

We analysed data from two ongoing national biologic cohort studies of children and young people with juvenile idiopathic arthritis in the UK: the British Society for Paediatric and Adolescent Rheumatology Etanercept Cohort Study (BSPAR-ETN),^11^ established 2004, and the Biologics for Children with Rheumatic Diseases (BCRD) study,^3^ established in 2010. Both studies use identical methodology, and patients can be switched between cohorts on the basis of the biologics received. Patients are tracked through the two studies to ensure all data can be combined and analysed for each unique patient.

Children and young people (aged <16 years) with physician-diagnosed juvenile idiopathic arthritis, classified according to the International League of Associations for Rheumatology (ILAR) criteria^12^ are eligible for inclusion in the studies and are recruited at the start of biologic therapy. Patients were not required to be biologic naive. We included all children and young people enrolled into BCRD or BSPAR-ETN between Jan 1, 2004, and April 11, 2019.

All participants or their legal guardians provided written informed consent in accordance with the Declaration of Helsinki. BSPAR-ETN was approved by the West Midlands Research Ethics Committee and BCRD was approved by the North West 7 REC Greater Manchester Central Ethics Committee.

### Procedures

Baseline data were collected at the start of biological therapy, including patient demographics (age, gender-identity), ILAR category, disease activity including active joint count (ie, swelling not caused by bony enlargement), limited joint count (limited range of motion plus tenderness, pain, or heat), physician's global assessment of overall disease activity (assessed using a visual analogue scale [0–10 cm]), patient (or parent) global assessment of overall wellbeing (PtGE; assessed using a visual analogue scale [0–10 cm]), erythrocyte sedimentation rate, C-reactive protein concentration, pain (assessed using a visual analogue scale [0–10 cm]), functional ability (assessed using the Childhood Health Assessment Questionnaire [CHAQ]), previous and current conventional synthetic therapy, and history of uveitis. Follow-up data were obtained from patient medical records by the prescribing team and transferred to the study database via online web system at 6 months, 1 year, and annually thereafter, and included changes to disease activity, changes to antirheumatic therapies (including start dates, stop dates, and reasons for cessation of therapy), and adverse events. For patients who switched biologic therapy, an additional form was used to collect data on disease activity at the time of switch and 6 months after switching.

### Statistical analysis

We split the data analysis into two parts: assessment of biologic switching patterns in all children who started their first biologic from Jan 1, 2010, and assessment of response to a second biologic, in all children who started their first biologic from Jan 1, 2004.

All children who initiated treatment with their first biologic from Jan 1, 2010, onwards, were included in the first part of the analysis. We used this date for two reasons: children starting non-etanercept biologics as their first biologic were only recruited from 2010 (before 2010, the studies were limited to children starting etanercept only), and a previous analysis based on these cohorts^3^ has shown that the pattern of biologic prescribing has changed over time, not only with regard to the choice of biologic, with a shift towards more non-etanercept biologics for children with certain disease features (ie, those with uveitis, or systemic juvenile idiopathic arthritis), but also towards earlier use of biologics. Thus, this date is more reflective of current biologic prescribing in patients with juvenile idiopathic arthritis. Patients were censored on the date of last study follow-up, date of death, or April 11, 2019 (data analysis cutoff), whichever came first. We calculated the proportion of patients who switched biologics at least once, and median time from initiation of first biologic to the initiation of a second, third, and fourth biologic. All biologics currently available for the treatment of inflammatory arthritis in children and adults were included, regardless of whether they were licensed specifically for juvenile idiopathic arthritis. Patterns of biologic switching were also stratified by whether patients had systemic juvenile idiopathic arthritis.

For the second part of the analysis, we included all children who initiated treatment with their first biologic from Jan 1, 2004, onwards, and had polyarticular course juvenile idiopathic arthritis (extended oligoarthritis or polyarthritis [positive or negative for rheumatoid factor]) with no active uveitis at the time of initiation of their second biologic. We applied the inclusion criterion because we hypothesized that a diagnosis of active uveitis or systemic juvenile idiopathic arthritis would limit the choice of biologic. We used linear regression to assess the change in outcome variables (active joint count; limited joint count; physician's global assessment of overall disease activity, PtGE, CHAQ, pain, erythrocyte sedimentation rate, and C-reactive protein concentration) and Juvenile Arthritis Disease Activity Score assessed in 71 joints (JADAS-71;^13^ composite score of the active joint count, physician's global assessment of disease activity, PtGE, and normalised erythrocyte sedimentation rate) between the time of initiation of a second biologic and 6 months thereafter, accounting for baseline values. Additionally, we assessed the American College of Rheumatology Paediatric (ACR Pedi) 90 response^14^— ie, the proportion of patients who achieve at least 90% improvement in at least three of the six core outcome variables (with no more than one worsening by more than 30%)—and the proportion of patients who achieved minimal disease activity (defined as physician's global assessment score ≤3·4; PtGE score ≤2·1, and ≤1 active joint^15^). Change in core outcome variables, JADAS-71 score, and the proportion of patients who achieved ACR Pedi 90, and minimal disease activity at 6 months was compared between patients starting a second TNFi and those switching to an alternative class of biologic using linear regression (for core outcome variables and JADAS-71 score) or logistic regression, adjusting for propensity quintiles to account for confounding by indication.^16^ For the ACR Pedi 90 response and minimal disease activity outcomes, patients who stopped biologic therapy before measurement of the 6-month outcomes were classified as non-responders, with the exception of patients who stopped treatment due to remission (as reported by the clinician) who were classified as responders. We used Kaplan-Meier curves to assess the duration of treatment with a second biologic during the first 2 years of treatment after initiation of a second biologic, using propensity quintile adjusted Cox-regression to compare the treatment duration between patients starting a second TNFi and those switching to another class of biologic. Reason for cessation of treatment with a second biologic by 2 years was compared between patients on a second TNFi and those on an alternative class of biologic using χ^2^ tests. We did two sensitivity analyses comparing changes in outcome variables, JADAS-71, and the proportion of patients achieving ACR Pedi 90 and minimal disease activity: the first analysis was limited to patients who initiated treatment with a second TNFi or tocilizumab after failure of an initial TNFi, and the second was limited to only patients who stopped treatment with their initial TNFi due to ineffectiveness.

We used multiple imputation (83 iterations based on the proportion of incomplete cases^17^) to account for missing data. We calculated propensity scores and stratified them into quintiles to include as an indicator variable in the regression models. The following variables included in the propensity score were measured at the time of initiation of a second biologic: second biologic start year (before 2010, 2010–15, or 2016–18), time since initiation of first biologic, gender, ILAR category, age, disease duration, concomitant methotrexate, concomitant steroids, active joint count, limited joint count, physician's global assessment, PtGE, CHAQ, pain, erythrocyte sedimentation rate, c-reactive protein concentration, and JADAS-71 score.

We used Stata software (version 14.0) for all analyses.

### Role of the funding source

The funders of the study had no role in study design, data collection, data analysis, data interpretation, or writing of the report. The corresponding author had full access to all the data in the study and had final responsibility for the decision to submit for publication.

## Results

Between Jan 1, 2004, and April 11, 2019, 2361 patients were enrolled on initiation of biologic therapy.

1152 patients (1055 with juvenile idiopathic arthritis; 97 with systemic juvenile idiopathic arthritis) initiated treatment with their first biologic from Jan 1, 2010, onwards, of whom 1081 (95%; of 1132 patients with available data) reported previous treatment with methotrexate. 1050 (91%) of 1152 patients started initial treatment with a TNFi, although among the 97 patients with systemic juvenile idiopathic arthritis, IL-6 and IL-1 inhibitors were the most common first biologic therapy (55 [57%] patients initiated treatment with the IL-6 inhibitor tocilizumab; 28 [29%] patients initiated treatment with an IL-1 inhibitor). A total of 2988 person-years of observed follow-up was available and the median duration of follow-up per patient was 2·2 years (IQR 1·1–3·8; maximum 9·1 years). During follow-up, 56 (5%) of 1152 patients withdrew or were lost to follow-up and 137 (12%) moved to an adult clinic where data capture from the adult hospital had not yet been established. 270 (23%) of 1152 patients started a second biologic after a median time of 1·3 years (IQR 0·6–2·3) from initiation of the first biologic, 61 (5%) patients started a third biologic after a median time of 2·5 years (IQR 1·6–3·7) from initiation of the first biologic, and 11 (1%) patients started a fourth biologic after a median time of 3·7 years (IQR 2·4–5·1) from initiation of the first biologic.

**Figure 1:**
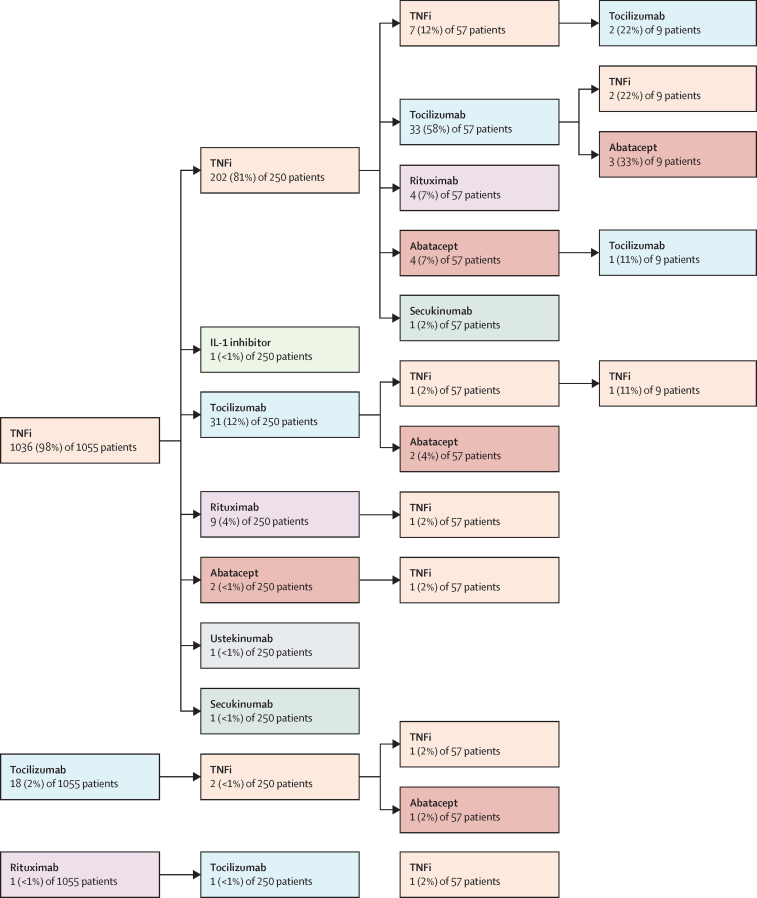
Biologic switching in patients with juvenile idiopathic arthritis (excluding systemic juvenile idiopathic arthritis) who initiated treatment with a first biologic from Jan 1, 2010, onwards (n=1055) TNFi=tumour necrosis factor inhibitor. IL=interleukin.

**Figure 2:**
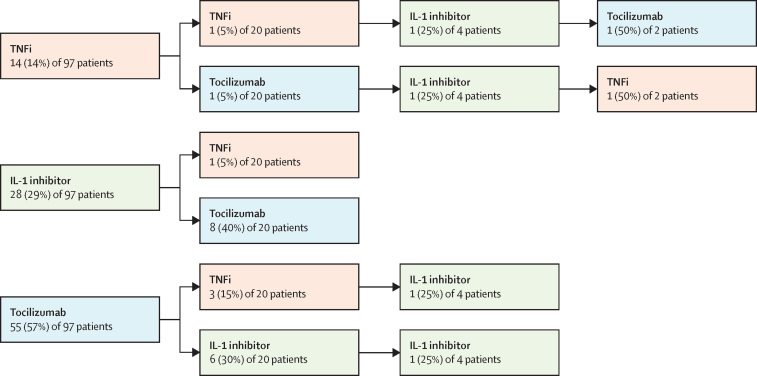
Biologic switching in patients with systemic juvenile idiopathic arthritis who started treatment with a first biologic from Jan 1, 2010, onwards (n=97) TNFi=tumour necrosis factor inhibitor. IL=interleukin.

Of the 270 patients who started a second biologic, 163 (60%) switched due to ineffectiveness and 66 (24%) switched due to adverse events (other reason or data missing for 41 [15%] patients). Of 1055 patients without systemic juvenile idopathic arthritis disease, 250 patients started a second biologic. 247 (99%) of these 250 patients had initiated treatment with a first TNFi biologic, of whom 202 (82%) started a second TNFi, whereas 45 (18%) switched from a TNFi to another class of biologic (figure 1). Of the 20 patients with systemic juvenile idiopathic arthritis who started a second biologic, eight (40%) patients started tocilizumab following initial treatment with an IL-1 inhibitor and six (30%) initiated treatment with an IL-1 inhibitor after initial treatment with tocilizumab (figure 2).

Of the 61 patients who started a third biologic, 24 (39%) switched due to repeated ineffectiveness, and 20 (33%) switched due to ineffectiveness and adverse events (other reason or data missing for 17 [28%] patients). Of the 57 patients without systemic juvenile idiopathic arthritis, 33 (58%) started tocilizumab, four (7%) started abatacept, and four (7%) started rituximab following two previous TNFis, although seven (12%) patients switched to a third TNFi. Four (4%) patients with systemic juvenile idiopathic arthritis started a third biologic, all of which were IL-1 inhibitors.

Of the 11 patients who started a fourth biologic, four (36%) switched due to repeated ineffectiveness and six (55%) switched due to ineffectiveness and adverse events (other reason or data missing for one [9%] patient). Six (55%) of 11 patients started either their third or fourth class of biologic. Of the 270 patients who had been exposed to at least two different biologic therapies, 25 (9%) patients had re-tried a biologic they had previously been treated with.

2361 patients were exposed to biologic therapy since Jan 1, 2004, and thus were eligible for inclusion in the second part of our data analysis (assessment of response to a second biologic). Of these 2361 patients, 817 (35%) patients reported switching to a second biologic, of whom 282 (35%) had polyarticular course juvenile idiopathic arthritis (oligoarticular extended or polyarticular [positive or negative for rheumatoid factor]) and had outcome data available. 42 of these 282 patients had active uveitis and were excluded. Thus, 240 patients were included in the analysis of response to a second biologic: 194 (81%) of 240 started a second TNFi, and 46 (19%) started an alternative class of biologic (tocilizumab [n=33], abatacept [n=6], rituximab [n=6], and the IL-12 and IL-23 inhibitor ustekinumab [n=1]). Patient characteristics at the initiation of a second biologic were similar between those who were starting treatment with a second TNFi and those switching to another class of biologic (table 1), including the reason for discontinuation of the first biologic.

**Table 1:** Baseline characteristics of patients with polyarticular course juvenile idiopathic arthritis (extended oligoarthritis or polyarthritis [positive or negative for rheumatoid factor]) who started a second biologic following initial TNFi (n=240)

		**Second TNFi (n=194)**	**Other biologic (n=46)**
Gender			
	Female	153 (79%)	42 (91%)
	Male	41 (21%)	4 (9%)
ILAR disease category at initiation of first TNFi			
	Extended oligoarthritis	64 (33%)	10 (22%)
	Polyarthritis (negative for rheumatoid factor)	98 (51%)	27 (59%)
	Polyarthritis (positive for rheumatoid factor)	32 (16%)	9 (20%)
First TNFi			
	Etanercept	158 (81%)	30 (65%)
	Infliximab	10 (5%)	4 (9%)
	Adalimumab	26 (13%)	12 (26%)
Disease duration at time of initiation of first TNFi (years)		2 (1–5)*	2 (1–6)
Reason for cessation of first biologic			
	Ineffectiveness	117 (60%)	27 (59%)
	Adverse events (excluding uveitis) or intolerance	36 (19%)	7 (15%)
	Ineffectiveness and intolerance	23 (12%)	7 (15%)
	Patient decision (injection related)	9 (5%)	3 (7%)
	Other	9 (5%)	2 (4%)
Time since initiation of first TNFi (years)		1·1 (0·5–2·5)	1·0 (0·7–3·5)
Start year of second biologic			
	Before 2010	23 (12%)	1 (2%)
	2010–15	77 (40%)	19 (41%)
	2016–19	94 (48%)	26 (57%)
Age at initiation of second biologic (years)		12 (9–15)	12 (9–15)
Disease duration at time of initiation of second biologic (years)		4 (2–7)*	5 (2–8)
Second biologic			
	Etanercept	10 (5%)	0
	Infliximab	70 (36%)	0
	Adalimumab	114 (59%)	0
	Rituximab	0	6† (13%)
	Tocilizumab	0	33 (72%)
	Abatacept	0	6 (13%)
	Ustekinumab	0	1 (2%)
Concomitant steroids within 2 weeks of initiation of second biologic		52/152 (34%)	7/35 (20%)
Concomitant methotrexate		132 (68%)	30 (65%)
Active joint count (71 joints)			
	Patients with available data	176 (91%)	43 (93%)
	Median (IQR)	3 (1–6)	3 (1–9)
Limited joint count (71 joints)			
	Patients with available data	174 (90%)	43 (93%)
	Median (IQR)	2 (0–6)	2 (0–5)
Physician's global assessment of disease activity (0–10 cm VAS)			
	Patients with available data	131 (68%)	29 (63%)
	Median (IQR)	3 (2–4)	4 (2–6)
Patient's or parent's global assessment of wellbeing (0–10 cm VAS)			
	Patients with available data	130 (67%)	32 (70%)
	Median (IQR)	5 (1–6)	5 (2–7)
Childhood Health Assessment Questionnaire score (range 0–3)			
	Patients with available data (n)	134 (69%)	36 (78%)
	Median (IQR)	0·9 (0·3–1·6)	1·1 (0·3–1·6)
Pain (0–10 cm VAS)			
	Patients with available data (n)	128 (66%)	32 (70%)
	Median (IQR)	5 (2–7)	5 (2–7)
Erythrocyte sedimentation rate (mm/h)			
	Patients with available data (n)	159 (82%)	35 (76%)
	Median (IQR)	10 (5–19)	10 (5–21)
C-reactive protein concentration (mg/L)			
	Patients with available data (n)	170 (88%)	44 (96%)
	Median (IQR)	5 (4–7)	4 (1–5)
JADAS-71 score			
	Patients with available data	91 (47%)	18 (39%)
	Median (IQR)	11 (6–17)	12 (5–22)

TNFi=tumour necrosis factor inhibitor. ILAR=International League Against Rheumatism. VAS=visual analogue scale. JADAS-71=Juvenile Arthritis Disease Activity Score assessed in 71 joints. Data are n (%), median (IQR), or n/N (%), unless specified otherwise.

* Data were missing for three patients.

† Two of six patients were positive for rheumatoid factor.

At 6 months (median follow-up 0·56 years [IQR 0·45–0·75]), no differences were identified with regard to change in individual outcome variables or JADAS-71 score between patients who started a second TNFi and those who initiated treatment with an alternative class of biologic (table 2). Among the 240 patients with polyarticular course juvenile idiopathic arthritis, a total of 22% (95% CI 16–28) of patients achieved an ACR Pedi 90 response, and 29% (23–36) of patients achieved minimal disease activity. No differences were identified in the proportion of patients who achieved an ACR Pedi 90 response (odds ratio [OR] 2·5, 95% CI 0·8–7·9; p=0·11), or minimal disease activity (OR 1·6, 95% CI 0·6–3·8; p=0·33) between patients starting a second TNFi versus an alternative class of biologic, adjusted for propensity quintiles.

**Table 2:** 6-month outcomes of patients with polyarticular course juvenile idiopathic arthritis (extended oligoarthritis or polyarthritis [positive or negative for rheumatoid factor]) who started a second biologic (n=240)

	**Second TNFi (n=194)**	**Other biologic (n=46)**	**p value**
**Active joint count (71 joints)**			
Baseline	5·3 (0·5)	5·0 (0·9)	0·77
6 months	2·3 (0·3)	2·7 (0·8)	0·61
Difference*	−2·9 (0·5)†	−2·2 (1·0)†	..
Unadjusted β coefficient (95% CI)	−0·5 (−1·8 to 0·9)	1 (ref)	0·51
Propensity quintile adjusted β coefficient (95% CI)‡	−0·6 (−2·9 to 1·8)	1 (ref)	0·64
**Limited joint count (71 joints)**			
Baseline	4·5 (0·5)	3·8 (0·8)	0·47
6 months	3·3 (0·5)	2·4 (0·9)	0·43
Difference*	−1·2 (0·6)†	−1·4 (1·2)†	..
Unadjusted β coefficient (95% CI)	0·7 (−1·6 to 2·9)	1 (ref)	0·55
Propensity quintile adjusted β coefficient (95% CI)‡	0·2 (−2·6 to 3·0)	1 (ref)	0·89
**Physician's global assessment of disease activity (VAS 0–10 cm)**			
Baseline	3·1 (0·2)	3·8 (0·4)	0·11
6 months	1·8 (0·2)	2·5 (0·4)	0·081
Difference*	−1·4 (0·2)†	−1·3 (0·5)†	..
Unadjusted β coefficient (95% CI)	−0·6 (−1·4 to 0·2)	1 (ref)	0·17
Propensity quintile adjusted β coefficient (95% CI)‡	−0·7 (−1·7 to 0·3)	1 (ref)	0·16
**PtGE (VAS 0–10 cm)**			
Baseline	4·1 (0·2)	4·6 (0·5)	0·43
6 months	2·9 (0·2)	3·8 (0·6)	0·089
Difference*	−1·2 (0·3)†	−0·7 (0·6)†	..
Unadjusted β coefficient (95% CI)	−0·8 (−1·8 to 0·3)	1 (ref)	0·14
Propensity quintile adjusted β coefficient (95% CI)‡	−0·8 (−2·1 to 0·4)	1 (ref)	0·19
**CHAQ score (range 0–3)**			
Baseline	1·03 (0·07)	1·10 (0·13)	0·63
6 months	0·89 (0·07)	1·02 (0·15)	0·40
Difference*	−0·14 (0·06)†	−0·08 (0·14)†	..
Unadjusted β coefficient (95% CI)	−0·1 (−0·3 to 0·2)	1 (ref)	0·52
Propensity quintile adjusted β coefficient (95% CI)‡	−0·04 (−0·3 to 0·2)	1 (ref)	0·80
**Pain (VAS 0–10)**			
Baseline	4·6 (0·3)	4·5 (0·5)	0·89
6 months	3·6 (0·2)	3·9 (0·5)	0·58
Difference*	−1·0 (0·3)†	−0·6 (0·6)†	..
Unadjusted β coefficient (95% CI)	−0·3 (−1·4 to 0·7)	1 (ref)	0·51
Propensity quintile adjusted β coefficient (95% CI)‡	−0·4 (−1·7 to 0·8)	1 (ref)	0·50
**Erythrocyte sedimentation rate (mm/h)**			
Baseline	18 (1·7)	16 (3·5)	0·73
6 months	12 (1·1)	9 (2·4)	0·21
Difference*	−5·4 (1·6)†	−7·2 (3·7)†	..
Unadjusted β coefficient (95% CI)	2·7 (−1·8 to 7·2)	1 (ref)	0·24
Propensity quintile adjusted β coefficient (95% CI)‡	2·4 (−4·8 to 9·7)	1 (ref)	0·51
**C-reactive protein concentration (mg/L)**			
Baseline	14 (2·2)	6·9 (2·2)	0·10
6 months	7·6 (1·3)	4·2 (1·6)	0·20
Difference*	−6·5 (2·0)†	−2·6 (2·7)†	..
Unadjusted β coefficient (95% CI)	1·2 (−3·2 to 5·7)	1 (ref)	0·58
Propensity quintile adjusted β coefficient (95% CI)‡	0·9 (−6·6 to 8·4)	1 (ref)	0·82
**JADAS-71 score**			
Baseline	13 (0·8)	14 (1·4)	0·68
6 months	7·3 (0·6)	9·4 (1·3)	0·10
Difference*	−5·9 (0·8)†	−4·5 (1·6)†	..
Unadjusted β coefficient (95% CI)	−1·9 (−4·3 to 0·5)	1 (ref)	0·12
Propensity quintile adjusted β coefficient (95% CI)‡	−2·2 (−5·8 to 1·4)	1 (ref)	0·23
**Proportion of patients who achieved ACR Pedi 90 (%)**§			
6 months (95% CI)	24% (17 to 31)	13% (2 to 25)	..
Unadjusted OR (95% CI)	2·1 (0·7 to 6·2)	1 (ref)	0·17
Propensity quintile adjusted OR (95% CI)‡	2·5 (0·8 to 7·9)	1 (ref)	0·11
**Proportion of patients who achieved minimal disease activity (%)**§			
6 months (95% CI)	31% (23 to 38)	23% (9 to 37)	..
Unadjusted OR (95% CI)	1·5 (0·7 to 3·5)	1 (ref)	0·31
Propensity quintile adjusted OR (95% CI)‡	1·6 (0·6 to 3·8)	1 (ref)	0·33

Date are mean (SE) or %, unless otherwise stated. Multiple imputation (83 datasets) was used to account for missing data. TNFi=tumour necrosis factor inhibitor. VAS=visual analogue scale. PtGE=patient's or parent's global assessment of overall wellbeing. CHAQ=childhood health assessment questionnaire. JADAS-71=Juvenile Arthritis Disease Activity Score assessed in 71 joints. ACR=American College of Rheumatology. ACR Pedi 90=ACR paediatric criteria for 90% improvement. OR=odds ratio. ILAR=International League Against Rheumatism.

* From time of initiation of second biologic to 6 months thereafter, accounting for baseline values.

† Significant change in variable between baseline and 6 months (p<0·05).

‡ Propensity quintile adjusted for the following variables at the time of initiation of second biologic: second biologic start year (before 2010, 2010–2015, or 2016–2018), time since initiation of first biologic, gender, ILAR category, age, disease duration, concomitant methotrexate, concomitant steroids, active joint count, limited joint count, physician's global assessment of overall disease activity, patient (or parent) evaluation of overall wellbeing, CHAQ, pain, erythrocyte sedimentation rate, C-reactive protein concentration, and JADAS-71 score.

§ For ACR Pedi 90 and minimal disease activity outcomes, patients who stopped biologic therapy before the 6-month outcome measurements were completed were classified as non-responders and those who stopped because they had achieved remission were classified as responders.

**Figure 3:**
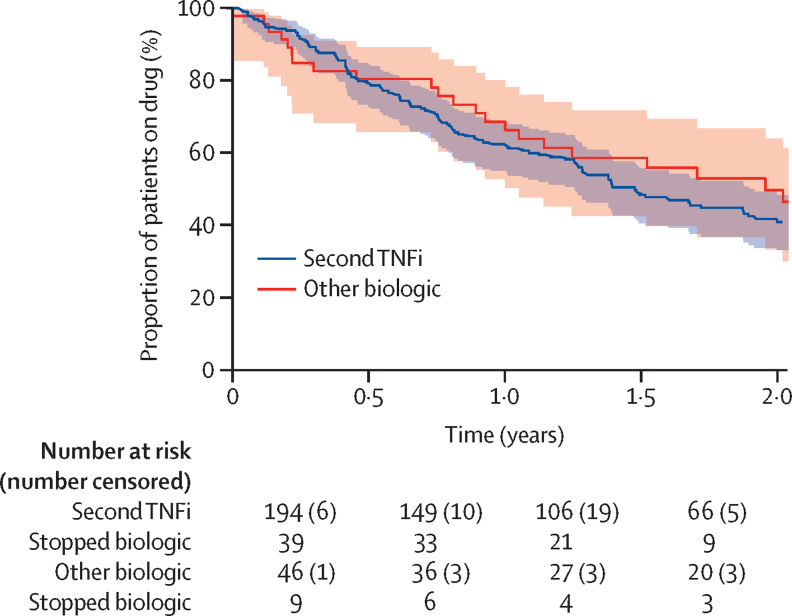
Kaplan-Meier analysis of duration of treatment in patients with polyarticular course juvenile idiopathic arthritis (extended oligoarthritis or polyarthritis [positive or negative for rheumatoid factor]) who started treatment with a second biologic after an initial TNFi (n=240) TNFi=tumour necrosis factor inhibitor. Patients were censored at the date of last clinic visit, analysis cutoff (April 11, 2019), or death, whichever came first, whichever came first. Shaded areas represent 95% CIs.

At 1 year, 63% (95% CI 55–68) of patients remained on their second biologic therapy and at 2 years, 42% (35–49) of patients remained on their second biologic therapy (figure 3). Propensity adjusted Cox-regression identified no significant differences in treatment duration with second biologic between patients on a second TNFi and patients on an alternative class of biologic (p=0·62). By 2 years, 55 (44%) of 124 patients had stopped their second biologic due to ineffectiveness and 17 (14%) due to adverse events, with no differences identified between the two cohorts.

Sensitivity analyses of patients who switched from their first biologic to a second TNFi or tocilizumab, and patients who started a second biologic due to ineffectiveness showed that at 6 months, no differences were identified in individual outcome variables or JADAS-71, or the proportion of patients who achieved ACR Pedi 90 or minimal disease activity between patients who initiated treatment with a second TNFi and those who initiated treatment with an alternative class of biologic in either analysis, adjusted for propensity quintiles (appendix pp 1–4).

## Discussion

To our knowledge, this is the first observational study to report on the extent of multibiologic switching in a cohort of children and young people with juvenile idiopathic arthritis, and to compare the effectiveness of different biologics after the failure of a first biologic. Biologic switching was common, with 23% of patients receiving at least two biologics, 5% receiving at least three, and 1% receiving at least four biologics during a median follow-up time of 2·2 years from initiation of their first biologic. However, there was no evidence that among children with polyarticular course juvenile idiopathic arthritis for whom treatment with a first biologic was ineffective, that switching classes of biologic was more beneficial than initiation of treatment with a second biologic within the same class.

Although the majority of patients with juvenile idiopathic arthritis tolerate biologic treatment well and have sustained disease control,^18^ some patients do not respond to, or cannot tolerate treatment. Estimating the proportion of patients with juvenile idiopathic arthritis who switch biologics multiple times allows appropriate service development and cost considerations. The use of data from a real-world cohort study is particularly valuable since these finding are representative of national prescribing patterns. The proportion of patients switching to a second biologic in our study was similar to that of a Dutch registry (26%),^7^ but the proportion of patients with systemic juvenile idiopathic arthritis who started a second biologic was lower than that in a French retrospective study (44%).^19^ The majority of patients recruited in the previous Dutch and French cohort studies started treatment with their first biologic before 2010 and therefore might not be representative of current biologic prescribing patterns. Whether a treat-to-target approach was specifically applied in some centres is unknown, thus it is unclear whether outcomes would be different if a specific treat-to-target guideline^20^, ^21^ was used, but is worth considering in future research whether such an approach was used and whether outcomes improve overall with such an approach. Patients who do not respond to biologic therapy are likely to require increased medical input compared with patients who respond to biologic therapy due to increased frequency of flares and increased need for drug education and monitoring.

In adults with rheumatoid arthritis, evidence supports switching from an initial TNFi to rituximab rather than a second TNFi.^22^ Evidence also indicates that in patients with exposure to at least one biologic, tocilizumab and a TNFi have similar effectiveness,^23^ as do rituximab and tocilizumab after initial treatment with a TNFi.^24^ However, rituximab is not a licensed treatment for children and young people with juvenile idiopathic arthritis and no formal controlled trials of rituximab in juvenile idiopathic arthritis have been done, thus the choice of approved biologics available remains limited. Most patients switch to a second TNFi, with many patients cycling onto their third TNFi despite the availability of alternative biologic therapies. The reasons for these choices are not known but the presence of uveitis might influence the choice of drug in some children.^3^ At present, two guidelines are available for biologic treatment of children and young people with juvenile idiopathic arthritis for whom an initial TNFi was ineffective. Both guidelines recommend contradictory treatment pathways: NHS England^5^ recommend patients switch to a second TNFi, whereas ACR^10^ recommend switching to a non-TNFi (tocilizumab or abatacept). Additionally, the NHS guidelines recommend that patients who are positive for rheumatoid factor should switch to rituximab instead of a TNFi, consistent with adult practice, whereas the ACR guidelines specifically recommend avoidance of this therapy, preferring the use of other classes primarily.

This study is a national cohort study and, although not mandatory, recruitment is recommended for all patients starting a biologic therapy^5^ and children have been recruited from almost every centre treating children with juvenile idiopathic arthritis in the UK. The study captures longitudinal prospective data from first biologic treatment, with the ability to track biologic switching. The use of robust statistical methods enabled the investigation of multiple outcomes in this analysis. However, juvenile idiopathic arthritis is a relatively rare disease with only 20% of patients estimated to start biologic therapy within the first 3 years after diagnosis,^25^ with one-fifth of these patients switching to a second biologic. Therefore, this analysis might not be powered to detect smaller differences in outcome, or differences between individual biologic therapies. The sensitivity analysis aimed to remove some of the heterogeneity from the other biologic drug classes, although whether these would be clinically meaningful is unknown. Patient numbers were further limited by the lack of additional core outcome data collected at the time of biologic switch and at 6 months because these forms were not introduced until 2014. A sensitivity analysis limited to patients who started a second biologic due to ineffectiveness of initial TNFi was done, however a similar analysis limited to children who stopped treatment with a first biologic due to an adverse event was not possible due to small patient numbers. Additionally, 42 patients had active uveitis at the time of initiation of their second biologic and thus were excluded, since it was unclear whether these patients were starting the biologic to treat their arthritis or their uveitis. The study did not capture any data on treatment adherence, drug levels, or anti-drug antibody concentrations, which might also influence treatment response. Furthermore, the route or frequency of biologic administration was not investigated, which might have also influenced treatment choice.

The time to initiation of a second biologic was more than 2·5 years after the initiation of a first TNFi in a quarter of children. For a rare disease such as juvenile idiopathic arthritis, it can take time to accumulate enough children for analysis and it is possible that outcomes might differ among children starting their second biologic in 2019, including quicker cycling through biologics. This reduction in time to treatment could in turn have resulted in better overall responses to a second biologic than those observed in the current study, although the proportion of adults with rheumatoid arthritis who respond well to a second biologic is lower than that with a first biologic.^22^

This is the first observational study to report that approximately one-fifth of children and young people with juvenile idiopathic arthritis in the UK starting their first biologic went on to receive a second biologic, and 5% received at least three biologics. Due to the frequent use of multiple TNFis, it is not possible to identify true multibiologic resistance in juvenile idiopathic arthritis, but the study shows that many children are being treated with multiple biologics. Additionally, among children with polyarticular course juvenile idiopathic arthritis for whom a first biologic was ineffective, no evidence was found to indicate that switching classes of biologic was more beneficial than initiation of treatment with a second biologic within the same class, despite current ACR guidelines.^10^ Ideally, a randomised trial comparing different second biologics could help address this question with more certainty. Further study of patients requiring multiple biologics is vital to enable patient specific treatment pathways, accurate prognosis discussions, and cost-effectiveness analysis for service provisions. Additional controlled trials of biologic medications are required in juvenile idiopathic arthritis because the number of approved therapeutic options remains small compared with those available for rheumatoid arthritis.

## References

[1] Ruperto N, Martini A (2011). Current medical treatments for juvenile idiopathic arthritis. Front Pharmacol.

[2] Minden K, Niewerth M, Zink A (2012). Long-term outcome of patients with JIA treated with etanercept, results of the biologic register JuMBO. Rheumatology.

[3] Kearsley-Fleet L, Davies R, Baildam E (2016). Factors associated with choice of biologic among children with juvenile idiopathic arthritis: results from two UK paediatric biologic registers. Rheumatology.

[4] Ringold S, Weiss PF, Beukelman T (2013). 2013 update of the 2011 American College of Rheumatology recommendations for the treatment of juvenile idiopathic arthritis: recommendations for the medical therapy of children with systemic juvenile idiopathic arthritis and tuberculosis screening among children receiving biologic medications. Arthritis Rheum.

[5] NHS England Clinical commissioning policy statement: biologic therapies for the treatment of juvenile idiopathic arthritis. 2015. https://www.england.nhs.uk/commissioning/wp-content/uploads/sites/12/2015/10/e03pd-bio-therapies-jia-oct15.pdf.

[6] Kearsley-Fleet L, Davies R, De Cock D (2018). Biologic refractory disease in rheumatoid arthritis: results from the British Society for Rheumatology Biologics Register for Rheumatoid Arthritis. Ann Rheum Dis.

[7] Otten MH, Prince FH, Anink J (2013). Effectiveness and safety of a second and third biological agent after failing etanercept in juvenile idiopathic arthritis: results from the Dutch National ABC Register. Ann Rheum Dis.

[8] National Institute for Health and Care Excellence TA35: guidance on the use of etanercept for the treatment of juvenile idiopathic arthritis. https://www.nice.org.uk/guidance/ta35.

[9] National Institute for Health and Care Excellence (2011). TA238: tocilizumab for the treatment of systemic juvenile idiopathic arthritis. https://www.nice.org.uk/guidance/ta238.

[10] Ringold S, Angeles-Han ST, Beukelman T (2019). 2019 American College of Rheumatology/Arthritis Foundation guideline for the treatment of juvenile idiopathic arthritis: therapeutic approaches for non-systemic polyarthritis, sacroiliitis, and enthesitis. Arthritis Care Res.

[11] Davies R, Southwood TR, Kearsley-Fleet L, Lunt M, Hyrich KL (2015). Medically significant infections are increased in patients with juvenile idiopathic arthritis treated with etanercept: Results from the British Society for Paediatric and Adolescent Rheumatology Etanercept Cohort Study. Arthritis Rheumatol.

[12] Manners P (2004). International League of Associations for Rheumatology classification of juvenile idiopathic arthritis: second revision. J Rheumatol.

[13] Consolaro A, Ruperto N, Bazso A (2009). Development and validation of a composite disease activity score for juvenile idiopathic arthritis. Arthritis Rheum.

[14] Giannini EH, Ruperto N, Ravelli A, Lovell DJ, Felson DT, Martini A (1997). Preliminary definition of improvement in juvenile arthritis. Arthritis Rheum.

[15] Magni-Manzoni S, Ruperto N, Pistorio A (2008). Development and validation of a preliminary definition of minimal disease activity in patients with juvenile idiopathic arthritis. Arthritis Rheum.

[16] Hade EM, Lu B (2014). Bias associated with using the estimated propensity score as a regression covariate. Stat Med.

[17] White IR, Royston P, Wood AM (2011). Multiple imputation using chained equations: issues and guidance for practice. Stat Med.

[18] Romano M, Pontikaki I, Gattinara M (2014). Drug survival and reasons for discontinuation of the first course of biological therapy in 301 juvenile idiopathic arthritis patients. Reumatismo.

[19] Woerner A, Uettwiller F, Melki I (2015). Biological treatment in systemic juvenile idiopathic arthritis: achievement of inactive disease or clinical remission on a first, second or third biological agent. RMD Open.

[20] Hinze CH, Holzinger D, Lainka E (2018). Practice and consensus-based strategies in diagnosing and managing systemic juvenile idiopathic arthritis in Germany. Pediatr Rheumatol Online J.

[21] Ravelli A, Consolaro A, Horneff G (2018). Treating juvenile idiopathic arthritis to target: recommendations of an international task force. Ann Rheum Dis.

[22] Soliman MM, Hyrich KL, Lunt M, Watson KD, Symmons DP, Ashcroft DM (2012). Rituximab or a second anti-tumor necrosis factor therapy for rheumatoid arthritis patients who have failed their first anti-tumor necrosis factor therapy? Comparative analysis from the British Society for Rheumatology Biologics Register. Arthritis Care Res (Hoboken).

[23] Lauper K, Nordström DC, Pavelka K (2018). Comparative effectiveness of tocilizumab versus TNF inhibitors as monotherapy or in combination with conventional synthetic disease-modifying antirheumatic drugs in patients with rheumatoid arthritis after the use of at least one biologic disease-modifying antirheumatic drug: analyses from the pan-European TOCERRA register collaboration. Ann Rheum Dis.

[24] Gottenberg JE, Morel J, Perrodeau E (2019). Comparative effectiveness of rituximab, abatacept, and tocilizumab in adults with rheumatoid arthritis and inadequate response to TNF inhibitors: prospective cohort study. BMJ.

[25] Davies R, Carrasco R, Foster HE (2016). Treatment prescribing patterns in patients with juvenile idiopathic arthritis (JIA): analysis from the UK Childhood Arthritis Prospective Study (CAPS). Semin Arthritis Rheum.

