# Supplementary material for: Frequency of biologic switching and the outcomes of switching in children and young people with juvenile idiopathic arthritis: a national cohort study
Source: Lancet Rheumatol. 2020 Mar 9;2(4):e217–26. doi: 10.1016/S2665-9913(20)30025-4 (PMC7134528; doi:10.1016/S2665-9913(20)30025-4)
Supplement: Supplementary appendix [file mmc1.pdf]

THE LANCET
Rheumatology

Supplementary appendix

This appendix formed part of the original submission and has been peer reviewed. We post it as supplied by the authors.

Supplement to: Kearsley-Fleet L, Heaf E, Davies R, et al. Outcomes and their frequency following biologic switching in children and young people with juvenile idiopathic arthritis: a national cohort study. *Lancet Rheumatol* 2020; published online March 9. https://doi.org/10.1016/S2665-9913(20)30025-4.

Supplementary Table 1: Six month outcomes (median 0·56 years [IQR 0·45, 0·74]) of patients with polyarticular-course JIA (oligoarticular extended or polyarticular rheumatoid factor positive or negative) starting a second biologic [N=227]; patients starting second TNFi versus patients starting tocilizumab.

		Second TNFi N=194	Tocilizumab N=33	p-value
**Active Joint Count [71 joints]**				
	Baseline	5·3 (0·5)	5·4 (1·1)	P=0·90
	Six month	2·3 (0·3)	2·5 (0·8)	P=0·87
	Change[#]	-2·9 (0·5)*	-3·0 (1·0)*	-
Unadjusted	beta	-0·1 (-1·5, 1·3)	Reference.	P=0·90
Propensity quintile adjusted[##]	beta	-0·2 (-2·8, 2·3)	Reference.	P=0·86
**Limited Joint Count [71 joints]**				
	Baseline	4·5 (0·5)	3·7 (0·9)	P=0·51
	Six month	3·3 (0·5)	1·9 (0·6)	P=0·21
	Change[#]	-1·2 (0·6)*	-1·9 (1·0)*	-
Unadjusted	beta	1·2 (-1·0, 3·4)	Reference.	P=0·29
Propensity quintile adjusted[##]	beta	0·6 (-2·2, 3·4)	Reference.	P=0·66
**Physician Global Assessment of Disease Activity (PGA) [range 0-10]**				
	Baseline	3·1 (0·2)	3·7 (0·4)	P=0·24
	Six month	1·8 (0·2)	2·0 (0·4)	P=0·56
	Change[#]	-1·4 (0·2)*	-1·7 (0·5)*	-
Unadjusted	beta	-0·1 (-1·0, 0·8)	Reference.	P=0·78
Propensity quintile adjusted[##]	beta	-0·3 (-1·4, 0·8)	Reference.	P=0·54
**Parent/Patient Global Evaluation of Well-Being (PtGE) [range 0-10]**				
	Baseline	4·1 (0·2)	4·3 (0·5)	P=0·75
	Six month	2·9 (0·2)	3·5 (0·7)	P=0·33
	Change[#]	-1·2 (0·3)*	-0·8 (0·8)*	-
Unadjusted	beta	-0·5 (-1·7, 0·7)	Reference.	P=0·37
Propensity quintile adjusted[##]	beta	-0·5 (-2·0, 1·0)	Reference.	P=0·51
**Childhood Health Assessment Questionnaire (CHAQ) [range 0-3]**				
	Baseline	1·03 (0·07)	1·06 (0·15)	P=0·85
	Six month	0·89 (0·07)	0·98 (0·16)	P=0·61
	Change[#]	-0·14 (0·06)*	-0·08 (0·15)*	-
Unadjusted	beta	-0·1 (-0·3, 0·2)	Reference.	P=0·62
Propensity quintile adjusted[##]	beta	-0·01 (-0·3, 0·3)	Reference.	P=0·95
**Pain VAS [range 0-10]**				
	Baseline	4·6 (0·3)	4·4 (0·5)	P=0·83
	Six month	3·6 (0·2)	3·6 (0·6)	P=0·92
	Change[#]	-1·0 (0·3)*	-0·8 (0·7)*	-
Unadjusted	beta	-0·1 (-1·3, 1·0)	Reference.	P=0·84
Propensity quintile adjusted[##]	beta	-0·2 (-1·6, 1·2)	Reference.	P=0·81
**ESR [mm/hr]**				
	Baseline	18 (1·7)	13 (3·0)	P=0·23
	Six month	12 (1·1)	5 (4·2)	P=0·018
	Change[#]	-5·4 (1·6)*	-6·7 (3·3)*	-
Unadjusted	beta	4·9 (0·1, 9·6)	Reference.	P=0·047
Propensity quintile adjusted[##]	beta	3·6 (-3·8, 11)	Reference.	P=0·34
**CRP [mg/l]**				
	Baseline	14 (2·2)	5·6 (2·0)	P=0·091
	Six month	7·6 (1·3)	3·4 (1·1)	P=0·14
	Change[#]	-6·5 (2·0)*	-2·2 (2·3)*	-
Unadjusted	beta	1·7 (-3·0, 6·4)	Reference.	P=0·48
Propensity quintile adjusted[##]	beta	0·9 (-7·0, 8·8)	Reference.	P=0·83
**JADAS-71**				
	Baseline	13 (0·8)	14 (1·7)	P=0·77
	Six month	7.3 (0·6)	8·2 (1·3)	P=0·52
	Change[#]	-5·9 (0·8)*	-5·6 (1·7)*	-
Unadjusted	beta	-0·8 (-3·4, 1·9)	Reference.	P=0·57
Propensity quintile adjusted[##]	beta	-1·1 (-5·1, 2·9)	Reference.	P=0·58

**ACR Pedi 90[a], %**				
All patients = 23%	Six month	24%	16%	-
Unadjusted, OR (95% CI)	-	1·6 (0·5, 5·1)	Reference.	P=0·39
Propensity quintile adjusted[##], OR (95% CI)	-	1·8 (0·6, 5·9)	Reference.	P=0·31
**Minimal Disease Activity[a], %**				
All patients = 30%	Six month	31%	25%	-
Unadjusted, OR (95% CI)	-	1·3 (0·5, 3·3)	Reference.	P=0·55
Propensity quintile adjusted[##], OR (95% CI)	-	1·4 (0·5, 3·7)	Reference.	P=0·50

*Tumour Necrosis Factor inhibitor (TNFi), Erythrocyte Sedimentation Rate (ESR), 71-joint Juvenile Arthritis Disease Activity Score (JADAS-71), American College of Rheumatology Paediatric criteria for improvement (ACR Pedi), Odds Ratio (OR), Confidence Interval (CI), International League Against Rheumatism (ILAR), Visual Analog Scale (VAS). Using imputed data (83 datasets). Results displayed as mean (standard error), unless otherwise stated. [a]Patients who stopped biologic therapy prior to the outcome measurements were classified as non-responders, or responders for those stopping for remission, for ACR Pedi 90 and MDA outcomes. #Change in variable taking into account baseline variable. *Significant change in variable from baseline to six months (p<0·05). ##Propensity quintile adjusted, included variables at start of second biologic: second biologic start year (<2010 / 2010-2015 / 2016-2018), time since first biologic, gender, ILAR category, age, disease duration, concomitant methotrexate, concomitant steroids, active joint count, limited joint count, physician global assessment of overall disease activity, patient (parent) evaluation of overall well-being, Childhood Health Assessment Questionnaire (CHAQ), pain VAS, ESR, CRP, JADAS-71.*

Supplementary Table 2: Six month outcomes (median 0·59 years [IQR 0·48, 0·75]) of patients with polyarticular-course JIA (oligoarticular extended or polyarticular rheumatoid factor positive or negative) starting a second biologic following ineffectiveness of their initial TNF inhibitor [N=144]; patients starting second TNF inhibitor versus patients starting a non-TNF inhibitor.

		Second TNFi N=117	Other Biologic N=27	p-value
**Active Joint Count [71 joints]**				
	Baseline	5·5 (0·7)	6·2 (1·3)	P=0·66
	Six month	2·4 (0·4)	2·7 (0·9)	P=0·79
	Change[#]	-3·1 (0·7)*	-3·5 (1·2)*	-
Unadjusted	beta	-0·1 (-1·8, 1·6)	Reference.	P=0·91
Propensity quintile adjusted[##]	beta	-0·3 (-3·4, 2·8)	Reference.	P=0·93
**Limited Joint Count [71 joints]**				
	Baseline	4·9 (0·7)	4·5 (1·2)	P=0·80
	Six month	3·8 (0·7)	2·4 (0·7)	P=0·36
	Change[#]	-1·1 (0·8)*	-2·1 (1·4)*	-
Unadjusted	beta	1·2 (-1·5, 4·0)	Reference.	P=0·38
Propensity quintile adjusted[##]	beta	0·6 (-3·1, 4·3)	Reference.	P=0·74
**Physician Global Assessment of Disease Activity (PGA) [range 0-10]**				
	Baseline	3·4 (0·2)	4·0 (0·5)	P=0·24
	Six month	2·0 (0·2)	2·4 (0·5)	P=0·48
	Change[#]	-1·4 (0·3)*	-1·7 (0·6)*	-
Unadjusted	beta	-0·2 (-1·3, 0·8)	Reference.	P=0·66
Propensity quintile adjusted[##]	beta	-0·5 (-1·8, 0·8)	Reference.	P=0·43
**Parent/Patient Global Evaluation of Well-Being (PtGE) [range 0-10]**				
	Baseline	4·2 (0·3)	4·8 (0·6)	P=0·44
	Six month	2·9 (0·3)	3·5 (0·7)	P=0·42
	Change[#]	-1·4 (0·4)*	-1·3 (0·8)*	-
Unadjusted	beta	-0·4 (-1·7, 1·0)	Reference.	P=0·59
Propensity quintile adjusted[##]	beta	-0·4 (-2·0, 1·2)	Reference.	P=0·58
**Childhood Health Assessment Questionnaire (CHAQ) [range 0-3]**				
	Baseline	1·00 (0·09)	1·19 (0·16)	P=0·32
	Six month	0·89 (0·08)	1·13 (0·18)	P=0·23
	Change[#]	-0·11 (0·07)*	-0·06 (0·17)	-
Unadjusted	beta	-0·1 (-0·4, 0·2)	Reference.	P=0·49
Propensity quintile adjusted[##]	beta	-0·1 (-0·4, 0·3)	Reference.	P=0·75
**Pain VAS [range 0-10]**				
	Baseline	4·8 (0·3)	5·0 (0·6)	P=0·84
	Six month	3·7 (0·3)	3·8 (0·7)	P=0·94
	Change[#]	-1·1 (0·3)*	-1·2 (0·7)*	-
Unadjusted	beta	-0·01 (-1·3, 1·3)	Reference.	P=0·99
Propensity quintile adjusted[##]	beta	-0·3 (-1·9, 1·3)	Reference.	P=0·71
**ESR [mm/hr]**				
	Baseline	20 (2·4)	15 (3·3)	P=0·29
	Six month	12 (1·6)	8·1 (3·2)	P=0·15
	Change[#]	-7·3 (2·4)*	-6·7 (3·0)	-
Unadjusted	beta	3·2 (-2·8, 9·1)	Reference.	P=0·29
Propensity quintile adjusted[##]	beta	2·7 (-6·7, 12)	Reference.	P=0·57
**CRP [mg/l]**				
	Baseline	13 (2·7)	8·1 (3·4)	P=0·32
	Six month	6·8 (1·3)	4·2 (1·3)	P=0·27
	Change[#]	-6·5 (2·6)*	-3·9 (3·5)*	-
Unadjusted	beta	1·7 (-2·5, 6·0)	Reference.	P=0·43
Propensity quintile adjusted[##]	beta	1·3 (-8·6, 11)	Reference.	P=0·80
**JADAS-71**				
	Baseline	14 (1·0)	15 (2·0)	P=0·53
	Six month	7.6 (0·7)	8·8 (1·5)	P=0·47
	Change[#]	-6·3 (1·1)*	-6·6 (1·9)*	-
Unadjusted	beta	-0·8 (-4·0, 2·3)	Reference.	P=0·59

Propensity quintile adjusted[##]	beta	-1·5 (-6·2, 3·2)	Reference.	P=0·54
**ACR Pedi 90[a], %**				
All patients = 22%	Six month	24%	13%	-
Unadjusted, OR (95% CI)	-	2·2 (0·5, 9·1)	Reference.	P=0·27
Propensity quintile adjusted[##], OR (95% CI)	-	2·9 (0·7, 13)	Reference.	P=0.16
**Minimal Disease Activity[a], %**				
All patients = 30%	Six month	31%	27%	-
Unadjusted, OR (95% CI)	-	1·2 (0·4, 3·4)	Reference.	P=0·73
Propensity quintile adjusted[##], OR (95% CI)	-	1·2 (0·4, 3·6)	Reference.	P=0·77

*Tumour Necrosis Factor inhibitor (TNFi), Erythrocyte Sedimentation Rate (ESR), 71-joint Juvenile Arthritis Disease Activity Score (JADAS-71), American College of Rheumatology Paediatric criteria for improvement (ACR Pedi), Odds Ratio (OR), Confidence Interval (CI), International League Against Rheumatism (ILAR), Visual Analog Scale (VAS). Using imputed data (83 datasets). Results displayed as mean (standard error), unless otherwise stated. [a]Patients who stopped biologic therapy prior to the outcome measurements were classified as non-responders, or responders for those stopping for remission, for ACR Pedi 90 and MDA outcomes. #Change in variable taking into account baseline variable. *Significant change in variable from baseline to six months (p<0·05). ##Propensity quintile adjusted, included variables at start of second biologic: second biologic start year (<2010 / 2010-2015 / 2016-2018), time since first biologic, gender, ILAR category, age, disease duration, concomitant methotrexate, concomitant steroids, active joint count, limited joint count, physician global assessment of overall disease activity, patient (parent) evaluation of overall well-being, Childhood Health Assessment Questionnaire (CHAQ), pain VAS, ESR, CRP, JADAS-71.*

Appendix: List of collaborators for the Biologics for Children with Rheumatic Diseases (BCRD) study, and the British Society for Paediatric and Adolescent Rheumatology Etanercept Cohort Study (BSPAR-ETN).

Principal Investigator	Centre
**Dr Kate Armon**	Addenbrookes Hospital
**Dr Michael Beresford**	Alder Hey Hospital, Liverpool
**Dr Liza McCann**	Alder Hey, Royal Liverpool Children's NHS Trust
**Dr Gillian Baksh**	Ashford and St Peters Hospital
**Dr Nagui Gendi**	Basildon Hospital, Essex
**Dr Eslam Al-Abadi**	Birmingham Children's Hospital
**Dr Kirsty Haslam**	Bradford Royal Infirmary
**Dr A V Ramanan**	Bristol Royal Hospital for Children
**Dr Kate Martin**	Cheltenham General Hospital, Child Health Offices
**Dr Kathryn Harrison**	Coventry and Warwickshire UH
**Dr Bridget Oates**	Crosshouse Hospital, Kilmarnock
**Dr Vicky Ohlsson**	Derriford Hospital, Plymouth
**Dr Nick Wilkinson**	Evelina London Children's Hospital
**Dr Flora McErlane**	Great North Children's Hospital Newcastle
**Dr Clarissa Pilkington**	Great Ormond Street, London
**Dr Mark Wood**	Leeds General Infirmary
**Dr Sridhar Arani**	Leicester Royal Infirmary
**Dr Alice Chieng**	Manchester Children's Hospital
**Dr Karen Davies**	New Cross Hospital, Wolverhampton
**Dr Kate Armon**	Norfolk and Norwich University Hospital
**Dr Rachel Jeffery**	Northampton General Hospital
**Dr Satyapal Rangaraj**	Nottingham University Hospitals
**Dr Kathryn Bailey**	Nuffield Orthopaedic Centre, Oxford
**Dr Afraa Al-Sabbagh**	Peterborough City Hospital
**Fouz Rahmeh**	Poole Hospital
**Dr Joanne Borbone**	Queen Alexandra Hospital, Portsmouth
**Dr Gulshan Malik**	Royal Aberdeen Children's Hospital
**Dr Richard Bowker**	Royal Derby Hospital
**Dr Nigel Osborne**	Royal Devon and Exeter Hospital
**Dr Rachel Tattersall**	Royal Hallamshire Hospital
**Dr Jo Walsh**	Royal Hospital for Children, Glasgow
**Dr Mary Brennan**	Royal Hospital for Sick Children, Edinburgh
**Dr Richard Brough**	Royal Shrewsbury Hospital
**Dr Daniel Hawley**	Sheffield Children's Hospital
**Dr Alice Leahy**	Southampton General Hospital
**Dr Frances Borg**	Southend University Hospital
**Dr Peter Fowlie**	Tayside Children's Hospital
**Dr Debajit Sen**	University College Hospital London
**Dr Coziana Ciurtin**	University College London Hospital
**Dr Jeremy Camilleri**	University Hospital Wales, Cardiff
**Dr Jon Packham**	University Hospitals of North Midlands NHS Trust
**Dr Kristen Healy**	Victoria Hospital, NHS Fife
**Dr Jonathan Rabbs**	Worthing Hospital
